# Advancing Tissue Culture with Light-Driven 3D-Printed Microfluidic Devices

**DOI:** 10.3390/bios14060301

**Published:** 2024-06-08

**Authors:** Xiangke Li, Meng Wang, Thomas P. Davis, Liwen Zhang, Ruirui Qiao

**Affiliations:** Australian Institute for Bioengineering and Nanotechnology, The University of Queensland, Brisbane, QLD 4072, Australia

**Keywords:** microfluidics, 3D printing, tissue culture

## Abstract

Three-dimensional (3D) printing presents a compelling alternative for fabricating microfluidic devices, circumventing certain limitations associated with traditional soft lithography methods. Microfluidics play a crucial role in the biomedical sciences, particularly in the creation of tissue spheroids and pharmaceutical research. Among the various 3D printing techniques, light-driven methods such as stereolithography (SLA), digital light processing (DLP), and photopolymer inkjet printing have gained prominence in microfluidics due to their rapid prototyping capabilities, high-resolution printing, and low processing temperatures. This review offers a comprehensive overview of light-driven 3D printing techniques used in the fabrication of advanced microfluidic devices. It explores biomedical applications for 3D-printed microfluidics and provides insights into their potential impact and functionality within the biomedical field. We further summarize three light-driven 3D printing strategies for producing biomedical microfluidic systems: direct construction of microfluidic devices for cell culture, PDMS-based microfluidic devices for tissue engineering, and a modular SLA-printed microfluidic chip to co-culture and monitor cells.

## 1. Introduction

Microfluidic systems, which enable the manipulation of minute fluid volumes (10^−9^ to 10^−18^ L) within channels sized from tens to hundreds of microns, have garnered considerable attention in scientific research and application [1]. Microfluidics offers several distinct advantages. Microfluidics can reduce sample and reagent quantities for cost savings and provide exceptional resolution and sensitivity for precise control of microemulsion generation [2]. The diminutive dimensions of microfluidic channels, characterized by high surface-area-to-volume ratios, promote enhanced thermal homogeneity at the reaction site, facilitating swift heat transfer [3]. These benefits allow microfluidics to be applied in biomedical applications such as tissue engineering, DNA purification, PCR activity, and medical diagnostics including pregnancy monitoring and glucose measurement [4].

Microfluidic devices have significant advantages over traditional batch methods in tissue culture. Traditional methods usually suffer from low throughput, high reagent consumption, and limited microenvironment control. By contrast, microfluidic devices can reduce reagent usage, enhance control of the cellular microenvironment, and perform high-throughput experiments [5]. First, microfluidic devices allow precise control of various environmental parameters, such as nutrient delivery, oxygen content, pH, and shear stress [6]. These advantages allow researchers to design customized microenvironments that closely resemble specific tissues or organs in the body. With this precise control, researchers can better simulate physiological conditions and, thus, obtain more biologically meaningful experimental results. Second, microfluidic devices offer unique advantages for high-throughput screening, as they can construct multiple chambers or channels. These capabilities allow for parallel experiments and high-throughput screening of multiple conditions or samples [7], significantly increasing experimental efficiency and accelerating the process of research and drug development. In addition, microfluidic devices are conveniently miniaturized and automated. Due to their compact structure, experiments can be greatly scaled down, reducing the amount of reagents and samples used [8]. At the same time, these devices can be connected to an automation system to improve fluid handling, sample and data collection operations, and experiment repeatability. Microfluidic devices can also be integrated with sensors, imaging systems, and other analysis tools to achieve real-time monitoring and analysis of cell reactions and behaviors [9]. This integration allows researchers to collect dynamic and time-resolved data that can reveal important insights into complex biological phenomena, including precise spatial and temporal control of the delivery of signaling molecules, drugs, and other stimuli to cells and tissues as well as gradient effects, cell-to-cell interactions, and dynamic biological responses [10]. By creating physiologically relevant models that closely mimic in vivo conditions, these benefits allow for the study of cellular behavior, drug responses, and disease mechanisms in a controlled environment [11]. As such, microfluidic devices play a crucial role in advancing biomedical research and fostering innovation in tissue culture methodologies. However, persistent challenges impede microfluidics integration across diverse fields, such as complex sample preparation, external high-tech equipment, and high-resolution requirements [12,13]. These complexities collectively pose significant hurdles to attaining large-scale commercial production. 

Three-dimensional printing, also known as additive manufacturing, refers to the process of creating three-dimensional objects by adding materials layer by layer based on a digital model. Recent advancements in three-dimensional (3D) printing have profoundly impacted microfabrication, tissue engineering, drug delivery, and medical device development [14,15]. Notably, 3D printing techniques have been applied to manufacturing complex microfluidic devices. They offer a cost-effective alternative to the time-consuming molding process and cleanroom facility requirements. 

However, traditional 3D printing techniques such as Fused Deposition Modeling (FDM), which extrude thermoplastic filaments layer by layer to create objects, often involve elevated processing temperatures, presenting challenges in using sensitive materials and biocompatible substances. Since FDM printing has a relatively lower resolution than other 3D printing methods, it inevitably reduces precision in microfluidic structure fabrication. Additionally, printed objects by Selective Laser Sintering and Selective Laser Melting (SLM) exhibit surface roughness, which impacts microfluidic devices’ fluidic behavior [16]. Addressing these challenges is essential for broadening the potential uses and capabilities of 3D printing technologies in the field of microfluidics.

Light-driven 3D printing methods, which employ photocuring to solidify liquid resins, have emerged as a promising solution for fabricating microfluidic systems [17]. These techniques, including Stereolithography (SLA), Digital Light Processing (DLP), and Inkjet printing, offer high resolutions, mild operational conditions, rapid production rates, relatively smooth surfaces, and a wide selection of resins. These benefits make light-driven 3D printing particularly well-suited to fabricating microfluidic devices, which are expected to have high-resolution channels, optical transparency, smooth internal channel surfaces, and robust mechanical stability to tolerate high pressure [18,19]. The emergence of new technology has significantly enhanced 3D-printed microfluidic devices’ research and applications in the field of biomedicine, opening new pathways for innovation and practicality. 

In this review, we introduce the current progress of light-based 3D-printed microfluidics and discuss their implications for pivotal areas such as cell culture, tissue engineering, as well as the co-culturing and monitoring of cells.

## 2. Introduction of Microfluidics

**Figure 1:**
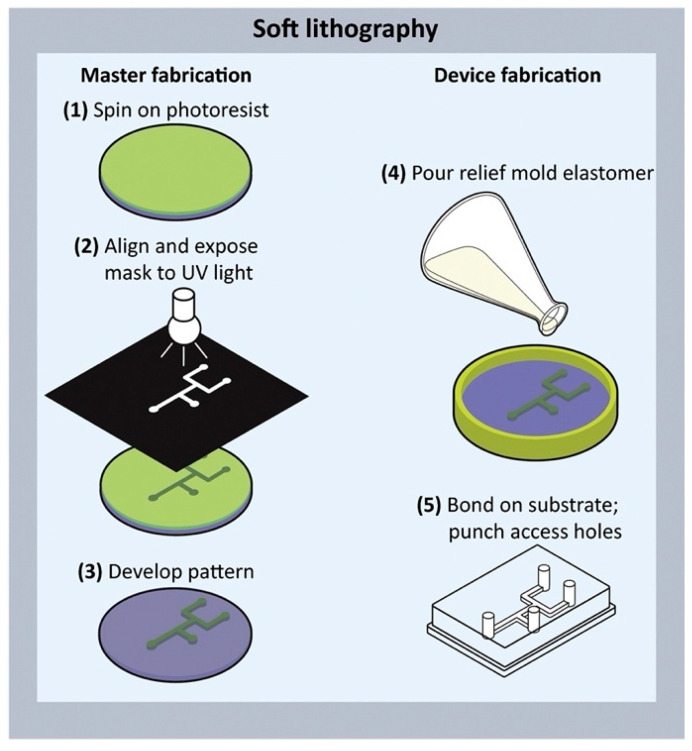
Rapid prototyping using soft lithography. Soft lithography is a multistep process in which a master mold is created, followed by curing a prepolymer substrate, peeling it off, bonding it to a substrate, and punching access holes [20]. Copyright 2017 Elsevier.

Microfluidic chips consist of a network of tiny channels etched or molded into materials such as siloxane and PDMS. They are also used for tasks such as mixing fluids, pumping, sorting particles, and regulating biochemical conditions [17]. These chips offer excellent gas permeability, surface modification capabilities, and biocompatibility, making them invaluable for assessing cell viability in vitro [20]. The transparency of microfluidic chips also allows for easier observation of fluid dynamics using optical microscopes [21]. Currently, soft lithography is the primary technique for fabricating microfluidic chips using elastomer masks, stamps, and molds for its rapid prototyping advantages [22,23]. Typically, photolithography is used to manufacture master molds for microfluidics with a pre-polymer such as PDMS cured on top of the mold. Once cured, a PDMS-negative stamp of the mold is created and permanently bonded to glass (Figure 1) [24].

These microfluidic chips can achieve high-resolution features at the micrometer and even nanometer scales. PDMS, a widely used polymer in microfluidics, is favored for its biocompatibility, ready availability, transparency, hydrophobic properties, gas permeability, and elastomeric nature [21]. As such, microfluidic chips manufactured through soft lithography are widely employed in the biological and medical fields, including genetic engineering, proteomics, medical diagnostics, cell culture, drug research and development, and biosensors for biochemical and pathogen detection [22]. However, soft lithography faces limitations related to material durability and chemical resistance. For instance, soft lithography-fabricated PDMS devices typically lack robustness, which can lead to flow profile issues due to leakage and uneven pressure distribution [25]. Additionally, PDMS has drawbacks such as lower mechanical robustness and chemical resistance than some 3D-printed resins [26,27]. In terms of mechanical characteristics, the elastic modulus of PDMS (measurement of stiffness) is between 1.32 and 2.97 MPa, and the tensile strength (stretch value before breaking) is between 3.51 and 5.13 MPa. These numbers can change depending on how much curing agent is used and the temperature at the time it was manufactured [28]. The absorption of small molecules by PDMS can also influence microfluidic experiments, especially in the areas of drug discovery, proteomic analysis, and cell culture, where the compounds being studied are present in very low concentrations [29]. Therefore, exploring other fabrication techniques becomes essential to overcoming these challenges and expanding microfluidic technology applications. Notably, 3D printing allows for rapid prototyping, scalability, and the production of complex geometries that are difficult to achieve with PDMS [30].

## 3. 3D-Printed Microfluidic Devices

Currently, 3D printing has become an advanced approach to creating microfluidic devices. Three-dimensional printing methods, such as FDM, PolyJet (PJ), SLA, etc., have been successfully employed to fabricate fluidic channels. Compared to soft lithography techniques, which have limited design complexity and customization, 3D printing offers faster prototyping and design flexibility for intricate and customized microfluidic devices.

**Figure 2:**
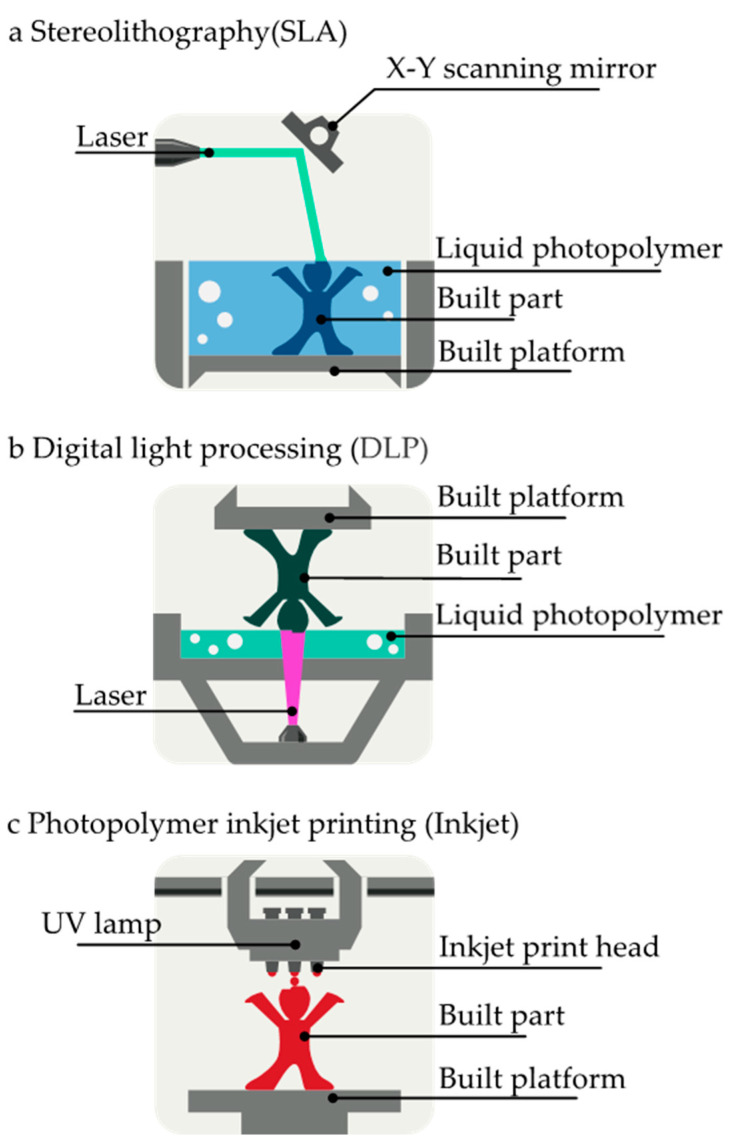
Schematic showing three types of light-induced 3D printing technologies.

SLA is one of the most widely used light-based 3D printing methods primarily due to its accessibility, swift printing capabilities, and production of smooth, accurate structures [31]. As shown in Figure 2a, this process involves the successive curing of liquid polymers layer by layer, employing laser UV light and a controlled built platform. The built platform plays a crucial role in supporting and positioning each layer of the object during printing, ensuring accuracy and structural integrity. At the same time, UV light allows precise liquid resin polymerization, solidifying it with each layer. This process results in a finely detailed and cohesive three-dimensional structure [32]. The UV laser performs two key functions in SLA printing: designing patterns and curing liquid resin. Generally, a UV laser source and a scanning mirror are used to design a raster pattern for printed models. Alternatively, the 2D pattern can be exposed to photo-curable resin using a UV source and a digital micromirror device to control printed shapes [33,34,35]. Using free surface or restricted surface methods for printing 3D materials is also feasible. Both methods involve the photopolymerization of liquid resins under UV light irradiation. An essential component in various SLA processes is an absorber, which reduces the light’s penetration into printed layers and prevents the polymerization of unpatented void features [13]. Hence, SLA’s precision and high-resolution output make it cost-effective for fabricating complex structures, especially microfluidic devices for tissue-related research [36,37,38,39]. L. Ding et al. used an SLA 3D printer to rapidly print modular microfluidic systems for detaching and separating mesenchymal stem cells (MSCs) from microcarriers (MCs) [40]. Direct SLA printing was used to create each module, resulting in inexpensive and easy-to-manufacture high-precision 3D objects [40].

DLP is another light-driven 3D printing technique using a digital light projector as a light source to cure photopolymers (Figure 2b) [41]. The UV light from the projector cures the photopolymer resin layer all at once, setting DLP apart from SLA where laser points trace each layer [42]. This simultaneous layer curing significantly accelerates printing speed while maintaining intricate designs and high accuracy. L. Wang et al. used DLP technology to manufacture polymer-based microfluidic chips, allowing for the rapid realization of a wide range of functional microstructures outside of a cleanroom and minimal masking requirements [43].

In photopolymer inkjet printing, inkjet print heads play a crucial role in expelling liquid photopolymers from built platforms. This material undergoes immediate curing and solidification upon exposure to UV lamps, facilitating layer-by-layer construction (Figure 2c) [44]. Photopolymer inkjet printing offers fewer constraints than alternative methods, making it highly suitable for creating patterned environments for drug-testing organoids and tissue models [45]. Lidia Donvito et al. developed an inkjet 3D-printed droplet microfluidic device from acrylonitrile and wax [46]. Inkjet printing eliminates misalignment issues that plague conventional manufacturing processes, making it possible to manufacture microfluidic chips in a single procedure. Moreover, it permits quick prototyping at minimal cost.

In summary, Table 1 provides a comparative overview that considers the operational principles, material usage, advantages, and limitations of three 3D printing techniques for microfluidic device fabrication.

## 4. Light-Driven 3D-Printed Microfluidics for Tissue Culture

In recent years, there has been a notable transformation in the methodologies employed for fabricating microfluidic devices. This change is attributed to the emergence of light-driven 3D printing, a new and promising method for overcoming some of the drawbacks of traditional soft lithography techniques [47]. Light-driven 3D printing offers an appealing option for creating microfluidic devices, mainly due to its cost-effectiveness, precision, high resolution, and ability to reproduce complex designs consistently [42,48]. Within the field of 3D-printed microfluidic devices, direct printing, mold-based, modular, and hybrid manufacturing procedures are the four primary production types. Microfluidic devices with integrated inlets, outlets, and microchannels are fabricated via 3D printing techniques. Novel techniques have also emerged, such as replica molding using 3D-printed molds. This approach involves the creation of microfluidic structures using 3D-printed molds, followed by bottom-side channel sealing with materials such as PDMS or glass slides. Another innovative concept is similar to assembling Lego^®^ blocks and involves piecing together 3D-printed microfluidic modules. Another notable advancement is a hybrid solution, where the lower layer of a 3D-printed micro-channel is connected to a transparent top layer, offering a unique combination of functionalities. The advantages of additive technology make 3D-printed microfluidics particularly useful in tissue engineering [47], including the construction of culture platforms. High-precision light-driven 3D printing allows microfluidics to become more readily available [49].

In tissue culture, 3D-printed microfluidic devices exhibit multiple functions and significantly enhance 3D cell culture techniques for various cell types, including mammalian cell lines, stem cells, and primary cells [50]. First, mammalian cell lines, such as HeLa cells and Chinese hamster ovary (CHO) cells, are widely used in biomedical research for applications as diverse as drug testing, gene expression studies, and cancer research. However, in traditional 2D cultures, these cells grow in monolayers, limiting their interaction with other cells and extracellular matrix components. By contrast, 3D-printed microfluidic devices provide a more complex environment that promotes better cell communication and function, thereby enhancing the physiological relevance of the studies conducted. Second, stem cells, including embryonic stem cells (ESCs) and induced pluripotent stem cells (iPSCs), greatly benefit from 3D culture systems. These cells require a highly controlled environment to maintain their pluripotency and differentiate them into specific cell types. Three-dimensionally printed microfluidic devices facilitate these complex cultures by providing controlled microenvironments and nutrient flow, which support the formation of multicellular spheroids and organoids and provide essential cues for differentiation and growth [51]. This level of control supports stem cell proliferation, differentiation, and the formation of complex tissue structures. It is especially critical for tissue engineering and regenerative medicine applications. In addition, primary cells that are directly isolated from tissue usually retain more of the physiological properties of their original tissue than immortalized cell lines. For instance, hepatocytes and cardiomyocytes benefit from the 3D architecture provided by these devices, which mimics in vivo conditions better than traditional 2D cultures. Specifically, hepatocytes are the primary functional cells of the liver and play a key role in drug metabolism and detoxification research. However, in 2D culture, liver cells often lose their phenotype and function very quickly. Three-dimensionally printed microfluidic devices can reconstruct the liver microenvironment, support liver cell function, and enable long-term studies of liver physiology and disease modeling [52].

Three-dimensional cell cultures, such as spheroids and organoids, offer a more physiologically relevant environment, promoting better tissue-like structures and functions [53]. Spheroids are simple cellular aggregates formed by a single cell type or a combination of multicellular cells typically grown as free-floating aggregates [54]. On the other hand, organoids are more sophisticated models created from iPSCs or by the self-organization of tissue-derived cells such as cancer cells or stem cells [54]. Organoids are highly complicated because the variety of cell types comprise their makeup better reflects the structure and function of the organ. Organoids are more complicated than spheroids and may be cultured for extended periods. Furthermore, 3D-printed microfluidic devices can be applied to organ-on-a-chip (OoCs) technologies, which simulate the functions of entire organs or organ systems on a micro-scale [51]. This model provides the scientific community with a cutting-edge tool by combining engineering and biological expertise. Therefore, 3D-printed microfluidic devices can replicate physiological conditions with high precision, offering a powerful tool for drug testing and disease modeling [55]. 

### 4.1. Light 3D-Printed Microfluidics for Spheroid Perfusion Culture

Spheroid perfusion culture is a cell cultivation method that involves growing three-dimensional cell clusters, or spheroids, in a controlled environment. In this technique, a continuous flow of culture media circulates through the spheroids, supplying nutrients and oxygen while removing waste products. This approach enhances cell viability and functionality, mimicking the natural tissue conditions more closely than traditional static cultures. Generally, microfluidic spheroid cultures use channels in microstructures to hold 3D cell clusters, enabling controlled nutrient perfusion for advanced cellular studies. This setup allows for the subsequent development of spheroids or organoids under medium perfusion conditions [56,57,58,59]. Since spheroids or organoids must be immobilized and fixed in microfluidic devices for extended periods ranging from days to weeks, monitoring downstream biological assays via visualization methods is necessary. Addressing these challenges requires careful consideration of biocompatibility, suitability for bioimaging, and facilitation of cell retrieval in the design and production of 3D-printed devices for tissue culture.

**Figure 3:**
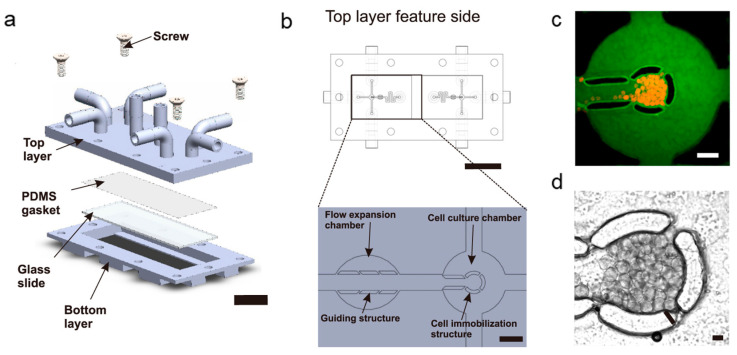
A 3D-printed microfluidic device for a spheroid culture system. (**a**) An exploded diagram of the device. (**b**) A 3D-printed top-layer device with microstructures for directing flow expansion and immobilizing cells (enlarged view). Imaging using fluorescence (**c**) and light transmission (**d**) showed spheroids of metastatic HN137 OSCC, which were trapped inside a cell culture chamber. To better view the cell culture chamber, FITC-tagged BSA was added to the culture media in (**c**). Scale bars: (**a**,**b**) 1 cm; (**b**) magnified view, 1 mm; (**c**) 500 μm; (**d**) 100 μm [60]. Copyright 2017 IOP Publishing.

In the context of spheroid cell culture and analysis, the unique operating principles and resolution restrictions of each 3D printing method are worth considering. To support multicellular spheroid cultures, Ong et al. reported the first example of a 3D-printed microfluidic perfusion cell culture device to directly immobilize 3D multicellular spheroids while keeping them alive and functioning [60]. They assessed two popular 3D printing technologies (SLA and PolyJet printing) to fabricate microfluidic devices containing cell-immobilized microstructures inside a microfluidic network. Their results revealed that SLA outperformed PolyJet printing, primarily in terms of resolution and post-processing ease. Notably, the 3D-printed microfluidic perfusion culture device comprised two separate components, a top layer, and a bottom mounting section (Figure 3a). Unlike a unibody device, this modular 3D-printed microfluidic device could be easily disassembled, allowing users to retrieve tissue samples without the need for specialized tools. In contrast to prototyping the device in a single build, this segmented design simplified the removal of support materials from the microfluidic network, making it possible to reuse the 3D-printed device. Notably, conventional 3D printing materials such as acrylonitrile butadiene styrene and polycarbonate exhibit limited transparency despite their prevalence [61,62,63]. This challenge was addressed by integrating a viewing window into the mounting base, which enables the visual examination of biological samples using sophisticated microscopy techniques, such as light and fluorescence microscopy. Additionally, delicately engineered PDMS membranes were introduced to serve as efficient gaskets during the integration of the top and mounting base components, ensuring the robust sealing of microfluidic channels. As depicted in Figure 3b, the schematic of the cell culture chamber’s central section features an array of microstructures arranged in a circular pattern. These microstructures physically entrap cells introduced via the seed intake channel. The cell entrapment method ensures that the spaces between these microstructures are smaller than the size of a single cell or cell aggregate, as documented in previous studies [56,64,65]. A different channel network was employed to facilitate the perfusion of the culture medium after the cells were seeded into the device. Notably, the cells entrapped within the device were cultured using a pump-free perfusion technique [66]. Furthermore, the entire 3D-printed microfluidic perfusion culture apparatus can be placed within a sterile secondary container and positioned within a controlled environment featuring a 37°C temperature and a CO_2_ incubator. This design enables the apparatus to function as a standalone device, eliminating the need for additional pumps and tubing. Figure 3c,d demonstrate the cell-immobilized microstructure array’s ability to effectively capture and retain both parental and metastatic oral squamous cell carcinoma (OSCC) tumor spheroids within the cell culture compartment for up to 72 h while maintaining high levels of viability and functionality. This microfluidic device, fabricated using SLA printing, facilitates the integration and substantial streamlining of the microfluidic culture system’s setup and operation. It broadens the system’s potential utility for investigating medication effectiveness, metabolic processes, and toxicity assessments.

### 4.2. Light 3D-Printed Replica Molding Process for Constructing Tissue-Engineered Skeletal Muscles

Functional skeletal muscle tissue substitutes show potential for treating a range of muscular diseases and injuries. Currently, photolithography, MultiJet Printing (MJP), and SLA are used to construct tissue-engineered skeletal muscles. Bian et al. manufactured polydimethylsiloxane (PDMS) molds via photolithography to create sizeable neonatal rat skeletal muscle tissue networks that exhibited consistent and controllable structural properties [67]. This technology requires costly cleanroom facilities, complex and error-prone procedures [68], and time-consuming post-processing [69]. Contrary to the photolithography method, Afshar et al. used a MJP plastic 3D printer to create a reusable mold, which was then used to produce a 96-well platform for large-scale production of 3D human skeletal muscle microtissues. Despite its effectiveness, MJP printing still has limitations related to the resolution of microfabrication techniques [70]. Its other limitations include high costs for high-end printers and slow, complex processes requiring specialized training, which restrict the broader adoption of these traditional fabrication methods in tissue engineering [71]. To address these challenges, employing readily available and affordable 3D printing materials, along with innovative replica molding techniques, are effective strategies for producing microdevices and scaling up at a low cost. Replica molding is a type of soft lithography for creating microfluidic chips by duplicating a mold’s morphology and structure [72]. Within a day, hundreds of 3D culture devices of varying sizes and geometries can be manufactured in-house using replica molding techniques. PBAT (Polybutylene Adipate Terephthalate) is fully biodegradable polyester. BASF, a leading manufacturer, produces PBAT under the Ecoflex^®^ (BASF, Ludwigshafen, Germany) brand name, which includes Ecoflex F Blend C1200 [73]. Ecoflex^®^, an economical, flexible, and highly resilient silicone material often employed in soft robotics, serves as a key component in this replica molding approach. The material is notable for its impressive stretchability, which can be stretched to over nine times its original length before breaking [74].

**Figure 4:**
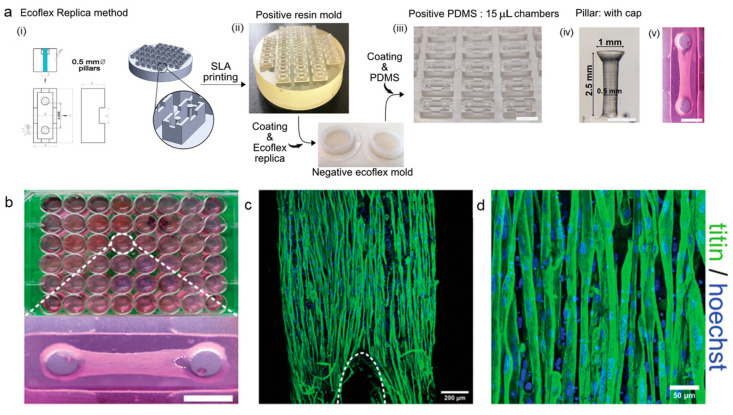
The Ecoflex^®^ Replica technique. Single-chamber technical drawing and 3D CAD model of the positive master mold (**i**); SLA-printed resin positive master mold (**ii**), Ecoflex^®^ negative mold replicas (**iii**), positive PDMS replica detail (**iii**), and T-shaped pillar with conical cap (**iv**). (**b**) TESMs grown in 15 µL Ecoflex^®^ Replica chambers were all contained on a 48-well plate. (**c**,**d**) Even in the smallest tissue, myogenic progenitors differentiated into long, multinucleated myofibers with an ordered titin pattern. The loop-like shape at the TESM’s far end, indicated by a dashed curved line, is the result of stress on the tissue. Scale bars: (**a**) 5 mm (**iii**); 1 mm (**iv**,**v**); (**b**) 1 mm; (**c**) 200 μm; (**d**) 50 μm [77]. Copyright 2020 Wiley.

The SLA printing technique can be used to fabricate replica moldings with smaller dimensions, excellent repeatability, and high throughput. Notably, SLA-printed objects exhibit both millimetric and micrometric features, a precision level difficult to achieve with conventional photolithography, particularly at the sub-millimeter scale [75,76]. To reproduce such structures in PDMS for high throughput applications of 3D culture devices, A. Iuliano et al. used the elastic polymer Ecoflex as a moldable replica substrate to construct tissue-engineered skeletal muscles (TESMS) in vitro [77]. The PBAT replica molding process was instrumental in generating negative molds following the initial 3D printing phase. Subsequently, the final positive PDMS structures could be effortlessly detached when the mold was stretched (Figure 4a). The creation of 3D culture chambers, featuring a volumetric capacity of 15 µL and T-shaped pillars measuring 500 µm in diameter and 2.5 mm in height, exemplifies a notable advancement in microfluidic device design. Most importantly, there is no discernible disparity between the original 3D-printed structures and PDMS replicas. Afterward, TESMs were cultivated in 48-well plates. Following 7 days of differentiation, engineered tissues in the 3D culture systems exhibited a consistent organization, characterized by the presence of long, aligned, and multinucleated myofibers positively stained for the sarcomeric protein titin. Confocal imaging revealed a typical striated titin pattern in the myofibers. The PBAT replica strategy has potential use in a wide range of applications due to its affordability and simplicity. It can be used as a compliant substrate for specific cell culture requirements and in developing supportive devices for other load-bearing tissues, such as the heart and tendons.

### 4.3. Light 3D-Printed Insert-Chip Microfluidics for Co-Culturing Cells

In recent years, in vitro modeling systems have been developed for mimicking cellular interactions. These modeling systems simulate the tissue microenvironment, illuminate human physiology, and exploit the underlying processes of disease [78,79]. To mimic in vivo microenvironments and barrier tissues, Transwell (TW) cell culture inserts are a conventional approach for investigating cell barriers that involves Transwell platforms, where cells are seeded on opposing sides of a porous polymer membrane [80,81,82,83]. Nevertheless, TW cell culture is static, making it challenging to develop models of vascular and epithelial tissues [84,85,86]. By contrast, microfluidic devices, known as “Organs-on-a-Chip” (OoCs), offer a distinctive approach by allowing the co-culturing of cells while maintaining controlled fluid flow—a departure from the static nature of TWs [87]. OoCs, essentially microfluidic chips containing biomimetic models of physiological organs, regulate fluid flow and offer valuable insights into interactions between different organs [88]. However, traditional OoCs are complex and integrated systems that involve time-consuming fabrication processes and demand specialized knowledge [9].

Currently, a transformative approach to overcoming limitations associated with TW inserts and conventional OoCs is using 3D-printed modular microfluidic systems [88]. Rauti et al. used modular structure-based SLA 3D printing techniques to customize microfluidic chips for co-culturing and monitoring various cell types under flow conditions. This work introduces a novel insert-chip, a microfluidic device with the functionalities of the OoCs platform, that facilitates cell co-culturing, exposure to flow, and observation of interactions [88]. These SLA-printed chips can be seamlessly integrated into standard cell culture platforms, including conventional well plate platforms such as microelectrode array platforms. Not only can these chips benefit from different technologies, but they can also cut down both development time and cost [89]. Compared to the laborious manufacturing steps of “conventional” OoCs, SLA-printed devices have their designs updated quickly to better suit their intended use and drastically decrease the manufacturing period from several days to a few hours. 

**Figure 5:**
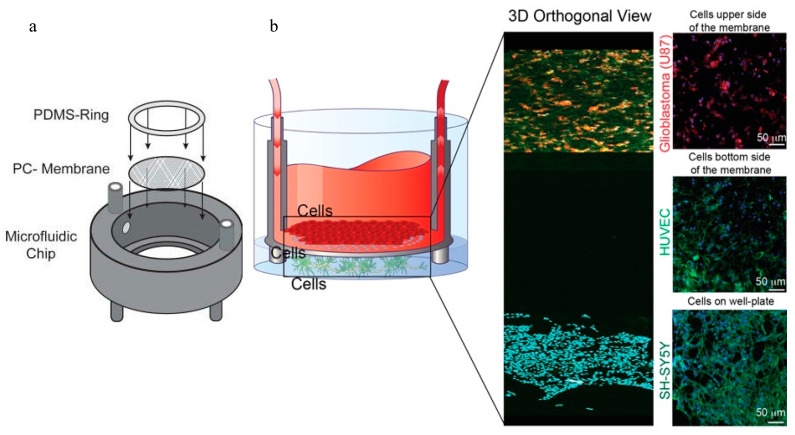
An insert-chip kind of layout. (**a**) An exploded look at the insert-chip reveals its three main parts: the 3D-printed base, the porous PC membrane, and the PDMS ring. (**b**) Tri-culture setup; the insert-chip can cultivate three distinct kinds of cells (on top of the membrane, at the bottom of the membrane, and at the bottom of the well). Three-dimensional confocal view of the system, with magnified images of SY-SH5Y (stained for actin in green and DAPI in blue) growing on the well-plate, U87 (stained for GFAP in red and DAPI in blue) on the top of the membrane, and HUVEC (stained for CD31 in green and DAPI in blue) on the bottom [88]. Copyright 2021 AIP Publishing.

The prevalent choice for fabricating microfluidic devices, such as OoCs, uses PDMS because of its acknowledged attributes of biocompatibility, transparency, and advantageous gas permeability [90]. However, a notable limitation of PDMS involves the absorption of hydrophobic substances [29]. Different from PDMS-manufactured chips, these 3D-printed chips are fabricated using non-absorbing materials. A transparent dental resin insert-chip was printed in the shape of a cylinder (Figure 5a) in which cells could grow over the porous membrane and the chip itself could store data. Each insert-chip has a cell culture chamber with a configurable exterior diameter of up to 25 mm, an internal diameter of 17 mm, and a medium capacity of up to 2 mL. The inlet and outlet channels of the chip’s upper portion enable connection to a regulated fluid flow system. These channels have external and internal channel dimensions of 2.5 mm and 1.5 mm, respectively. To overcome limitations related to high-magnification imaging on standard dual-channel OoCs, the researchers devised a membrane that can be extracted from the chip using tweezers (Figure 5a). Subsequently, the membrane can be placed on a glass coverslip for standard immunocytochemistry processes and microscopic examination. Figure 5b shows a confocal reconstruction with magnified images of SY-SH5Y, U87, and HUVEC from bottom to top. 

## 5. Summary

In this review, the definitions, theories, and advantages of three principal light-driven 3D printing technologies are provided: SLA, DLP, and photopolymer inkjet printing. We also discuss three specific strategies for using light-driven 3D printing to fabricate microfluidic devices, such as (1) utilizing SLA to directly print microfluidic device channels; (2) creating microfluidic devices using molds produced via SLA printing; (3) employing a modular approach to assemble microfluidic chips. Furthermore, the biomedical applications of these printed microfluidic devices are explored, including spheroid perfusion culture, the replica molding process for constructing tissue-engineered skeletal muscles, and insert-chip microfluidics designed to enhance cell co-cultures.

In the coming years, we anticipate that light-driven 3D printing technologies will be the primary choice for manufacturing microfluidic devices. However, materials and technical development must first achieve higher resolution, optimal optical properties, and enhanced biocompatibility. Although light-driven 3D printing offers benefits in manufacturing microfluidic devices, several limitations must be resolved. UV absorbers and photoinitiators in SLA and DLP resins may cause cytotoxicity, which has risks for cell-based applications. The type of resin selected significantly impacts cytotoxicity and gas permeability. Recent advancements in developing PDMS-based resins have led to improved low-viscosity resins that retain PDMS properties, such as optical clarity, gas permeability, and biocompatibility, enabling automated fabrication of microfluidic devices via 3D printing [91]. In addition, surface treatment methods can significantly enhance the optical quality of directly printed microfluidic devices [92]. Several methods have been used to enhance surface smoothness, including (1) mechanical surface treatments such as sand polishing [93]; (2) chemical polishing treatments [94]; (3) polymer coatings such as spray-coated clear acrylic [95].

While current DLP printers have high resolution, they fail to achieve the ultra-fine resolution required for fabricating intricate microchannels essential to microfluidic devices. Additionally, a significant technical challenge is controlling light penetration and exposure during the 3D printing process, which is vital for ensuring the quality and reliability of microfluidic channels. UV overexposure can cause channel blockages while inadequate curing time causes the leakage of photoinitiators and unreacted monomers. Unlike traditional SLA, TPP uses femtosecond laser pulses to initiate polymerization at the focal point, allowing for voxel-by-voxel construction of 3D structures at a sub-micron scale [96]. This process enables the creation of complex geometries with smooth surfaces and high aspect ratios below 100 nm precision, which are essential for precise microfluidic applications [97]. Therefore, incorporating TPP into microfluidics fabrication can significantly enhance the precision and functionality of these devices. As technology continues to advance, we can expect even more sophisticated and application-specific microfluidic devices to emerge, further pushing the boundaries of what is possible in tissue culture and biomedical research. Although light-driven 3D printing has not become mainstream in producing microfluidic chips, it is creating new opportunities for businesses and research institutions to significantly impact global healthcare.

## Figures and Tables

**Table 1:** Summary of three main light-driven 3D printing techniques for fabricating microfluidic devices.

3D Printing Technique	Energy Source	Materials	Advantages	Disadvantages
SLA	UV	Photocurable resin/polymer	Easy-to-make large pieces, allow uncured material, and high precision	Post-curing and support removal required
DLP	UV	Photocurable resin/polymer	Laying precision, high resolution, and reusing uncured photopolymer	Consumables insecurity and unsuitable for large structures
Inkjet	UV	Photocurable resin/polymer	Multi-material, fast-build printing	Laborious to remove channel support materials

## References

[1] Whitesides G.M. (2006). The origins and the future of microfluidics. Nature.

[2] Chiu Y.-L., Chan H.F., Phua K.K.L., Zhang Y., Juul S., Knudsen B.R., Ho Y.-P., Leong K.W. (2014). Synthesis of Fluorosurfactants for Emulsion-Based Biological Applications. ACS Nano.

[3] Dittrich P.S., Manz A. (2006). Lab-on-a-chip: Microfluidics in drug discovery. Nat. Rev. Drug Discov..

[4] Pattanayak P., Singh S.K., Gulati M., Vishwas S., Kapoor B., Chellappan D.K., Anand K., Gupta G., Jha N.K., Gupta P.K. (2021). Microfluidic chips: Recent advances, critical strategies in design, applications and future perspectives. Microfluid. Nanofluid..

[5] Velve-Casquillas G., Le Berre M., Piel M., Tran P.T. (2010). Microfluidic tools for cell biological research. Nano Today.

[6] Tehranirokh M., Kouzani A.Z., Francis P.S., Kanwar J.R. (2013). Microfluidic devices for cell cultivation and proliferation. Biomicrofluidics.

[7] De Stefano P., Bianchi E., Dubini G. (2022). The impact of microfluidics in high-throughput drug-screening applications. Biomicrofluidics.

[8] Lin C.-C., Wang J.-H., Wu H.-W., Lee G.-B. (2010). Microfluidic Immunoassays. J. Assoc. Lab. Autom..

[9] Coluccio M.L., Perozziello G., Malara N., Parrotta E., Zhang P., Gentile F., Limongi T., Raj P.M., Cuda G., Candeloro P. (2019). Microfluidic platforms for cell cultures and investigations. Microelectron. Eng..

[10] Cardoso B.D., Castanheira E.M.S., Lanceros-Mendez S., Cardoso V.F. (2023). Recent Advances on Cell Culture Platforms for In Vitro Drug Screening and Cell Therapies: From Conventional to Microfluidic Strategies. Adv. Healthc. Mater..

[11] Clancy A., Chen D., Bruns J., Nadella J., Stealey S., Zhang Y., Timperman A., Zustiak S.P. (2022). Hydrogel-based microfluidic device with multiplexed 3D in vitro cell culture. Sci. Rep..

[12] Battat S., Weitz D.A., Whitesides G.M. (2022). An outlook on microfluidics: The promise and the challenge. Lab Chip.

[13] Nielsen J.B., Hanson R.L., Almughamsi H.M., Pang C., Fish T.R., Woolley A.T. (2020). Microfluidics: Innovations in Materials and Their Fabrication and Functionalization. Anal. Chem..

[14] Pavan Kalyan B., Kumar L. (2022). 3D Printing: Applications in Tissue Engineering, Medical Devices, and Drug Delivery. AAPS PharmSciTech.

[15] Tasoglu S., Folch A. (2018). Editorial for the Special Issue on 3D Printed Microfluidic Devices. Micromachines.

[16] Arefin A.M.E., Khatri N.R., Kulkarni N., Egan P.F. (2021). Polymer 3D Printing Review: Materials, Process, and Design Strategies for Medical Applications. Polymers.

[17] Villegas M., Cetinic Z., Shakeri A., Didar T.F. (2018). Fabricating smooth PDMS microfluidic channels from low-resolution 3D printed molds using an omniphobic lubricant-infused coating. Anal. Chim. Acta.

[18] Yadavali S., Jeong H.-H., Lee D., Issadore D. (2018). Silicon and glass very large scale microfluidic droplet integration for terascale generation of polymer microparticles. Nat. Commun..

[19] Luo Z., Zhang H., Chen R., Li H., Cheng F., Zhang L., Liu J., Kong T., Zhang Y., Wang H. (2023). Digital light processing 3D printing for microfluidic chips with enhanced resolution via dosing-and zoning-controlled vat photopolymerization. Microsyst. Nanoeng..

[20] Walsh D.I., Kong D.S., Murthy S.K., Carr P.A. (2017). Enabling Microfluidics: From Clean Rooms to Makerspaces. Trends Biotechnol..

[21] Whitesides G.M., Ostuni E., Takayama S., Jiang X., Ingber D.E. (2001). Soft Lithography in Biology and Biochemistry. Annu. Rev. Biomed. Eng..

[22] Yeo L.Y., Chang H.-C., Chan P.P.Y., Friend J.R. (2011). Microfluidic Devices for Bioapplications. Small.

[23] Xia Y., Whitesides G.M. (1998). Soft Lithography. Angew. Chem. Int. Ed..

[24] Melin J., Quake S.R. (2007). Microfluidic Large-Scale Integration: The Evolution of Design Rules for Biological Automation. Annu. Rev. Biophys. Biomol. Struct..

[25] Kim K., Park S.W., Yang S.S. (2010). The optimization of PDMS-PMMA bonding process using silane primer. BioChip J..

[26] Ko Y.H., Lee S.H., Leem J.W., Yu J.S. (2014). High transparency and triboelectric charge generation properties of nano-patterned PDMS. RSC Adv..

[27] Lee J., Kim M. (2022). Polymeric Microfluidic Devices Fabricated Using Epoxy Resin for Chemically Demanding and Day-Long Experiments. Biosensors.

[28] Ariati R., Sales F., Souza A., Lima R.A., Ribeiro J. (2021). Polydimethylsiloxane Composites Characterization and Its Applications: A Review. Polymers.

[29] Toepke M.W., Beebe D.J. (2006). PDMS absorption of small molecules and consequences in microfluidic applications. Lab Chip.

[30] Wang X., Jiang M., Zhou Z., Gou J., Hui D. (2017). 3D printing of polymer matrix composites: A review and prospective. Compos. Part B Eng..

[31] O’Neill P.F., Ben Azouz A., Vázquez M., Liu J., Marczak S., Slouka Z., Chang H.C., Diamond D., Brabazon D. (2014). Advances in three-dimensional rapid prototyping of microfluidic devices for biological applications. Biomicrofluidics.

[32] Amin R., Knowlton S., Hart A., Yenilmez B., Ghaderinezhad F., Katebifar S., Messina M., Khademhosseini A., Tasoglu S. (2016). 3D-printed microfluidic devices. Biofabrication.

[33] Waldbaur A., Rapp H., Länge K., Rapp B.E. (2011). Let there be chip—Towards rapid prototyping of microfluidic devices: One-step manufacturing processes. Anal. Methods.

[34] Melchels F.P.W., Feijen J., Grijpma D.W. (2010). A review on stereolithography and its applications in biomedical engineering. Biomaterials.

[35] Grogan S.P., Chung P.H., Soman P., Chen P., Lotz M.K., Chen S., D’Lima D.D. (2013). Digital micromirror device projection printing system for meniscus tissue engineering. Acta Biomater..

[36] Utada A.S., Lorenceau E., Link D.R., Kaplan P.D., Stone H.A., Weitz D.A. (2005). Monodisperse double emulsions generated from a microcapillary device. Science.

[37] Lim T.W., Son Y., Jeong Y.J., Yang D.-Y., Kong H.-J., Lee K.-S., Kim D.-P. (2011). Three-dimensionally crossing manifold micro-mixer for fast mixing in a short channel length. Lab Chip.

[38] Nielson R., Kaehr B., Shear J.B. (2009). Microreplication and Design of Biological Architectures Using Dynamic-Mask Multiphoton Lithography. Small.

[39] Zhang L., Forgham H., Shen A., Wang J., Zhu J., Huang X., Tang S.-Y., Xu C., Davis T.P., Qiao R. (2022). Nanomaterial integrated 3D printing for biomedical applications. J. Mater. Chem. B.

[40] Ding L., Razavi Bazaz S., Asadniaye Fardjahromi M., Mckinnirey F., Saputro B., Banerjee B., Vesey G., Ebrahimi Warkiani M. (2022). A modular 3D printed microfluidic system: A potential solution for continuous cell harvesting in large-scale bioprocessing. Bioresour. Bioprocess..

[41] Zhang J., Hu Q., Wang S., Tao J., Gou M. (2020). Digital Light Processing Based Three-dimensional Printing for Medical Applications. Int. J. Bioprint.

[42] Prabhakar P., Sen R.K., Dwivedi N., Khan R., Solanki P.R., Srivastava A.K., Dhand C. (2021). 3D-Printed Microfluidics and Potential Biomedical Applications. Front. Nanotechnol..

[43] Wang L., Kodzius R., Yi X., Li S., Hui Y.S., Wen W. (2012). Prototyping chips in minutes: Direct Laser Plotting (DLP) of functional microfluidic structures. Sens. Actuators B Chem..

[44] Ho C.M.B., Ng S.H., Li K.H.H., Yoon Y.-J. (2015). 3D printed microfluidics for biological applications. Lab Chip.

[45] Tse C.C.W., Smith P.J., Ertl P., Rothbauer M. (2018). Inkjet Printing for Biomedical Applications. Cell-Based Microarrays: Methods and Protocols.

[46] Donvito L., Galluccio L., Lombardo A., Morabito G., Nicolosi A., Reno M. (2015). Experimental validation of a simple, low-cost, T-junction droplet generator fabricated through 3D printing. J. Micromech. Microeng..

[47] Mehta V., Rath S.N. (2021). 3D printed microfluidic devices: A review focused on four fundamental manufacturing approaches and implications on the field of healthcare. Bio-Des. Manuf..

[48] Lee J.M., Zhang M., Yeong W.Y. (2016). Characterization and evaluation of 3D printed microfluidic chip for cell processing. Microfluid. Nanofluid..

[49] Lerman M.J., Lembong J., Gillen G., Fisher J.P. (2018). 3D printing in cell culture systems and medical applications. Appl. Phys. Rev..

[50] Fang Y., Eglen R.M. (2017). Three-Dimensional Cell Cultures in Drug Discovery and Development. SLAS Discov..

[51] Li X.J., Valadez A.V., Zuo P., Nie Z. (2012). Microfluidic 3D cell culture: Potential application for tissue-based bioassays. Bioanalysis.

[52] Kapalczynska M., Kolenda T., Przybyla W., Zajaczkowska M., Teresiak A., Filas V., Ibbs M., Blizniak R., Luczewski L., Lamperska K. (2018). 2D and 3D cell cultures—A comparison of different types of cancer cell cultures. Arch. Med. Sci..

[53] Salinas-Vera Y.M., Valdes J., Perez-Navarro Y., Mandujano-Lazaro G., Marchat L.A., Ramos-Payan R., Nunez-Olvera S.I., Perez-Plascencia C., Lopez-Camarillo C. (2022). Three-Dimensional 3D Culture Models in Gynecological and Breast Cancer Research. Front. Oncol..

[54] Urzi O., Gasparro R., Costanzo E., De Luca A., Giavaresi G., Fontana S., Alessandro R. (2023). Three-Dimensional Cell Cultures: The Bridge between In Vitro and In Vivo Models. Int. J. Mol. Sci..

[55] Wu X., Shi W., Liu X., Gu Z. (2024). Recent advances in 3D-printing-based organ-on-a-chip. EngMedicine.

[56] Lee P.J., Hung P.J., Lee L.P. (2007). An artificial liver sinusoid with a microfluidic endothelial-like barrier for primary hepatocyte culture. Biotechnol. Bioeng..

[57] Bhushan A., Senutovitch N., Bale S.S., Mccarty W.J., Hegde M., Jindal R., Golberg I., Berk Usta O., Yarmush M.L., Vernetti L. (2013). Towards a three-dimensional microfluidic liver platform for predicting drug efficacy and toxicity in humans. Stem Cell Res. Ther..

[58] Tan G.-D.S., Toh G.W., Birgersson E., Robens J., Van Noort D., Leo H.L. (2013). A thin-walled polydimethylsiloxane bioreactor for high-density hepatocyte sandwich culture. Biotechnol. Bioeng..

[59] Wang Y., Toh Y.C., Li Q., Nugraha B., Zheng B., Lu T.B., Gao Y., Ng M.M., Yu H. (2013). Mechanical compaction directly modulates the dynamics of bile canaliculi formation. Integr. Biol..

[60] Ong L.J.Y., Islam A., Dasgupta R., Iyer N.G., Leo H.L., Toh Y.-C. (2017). A 3D printed microfluidic perfusion device for multicellular spheroid cultures. Biofabrication.

[61] Waheed S., Cabot J.M., Macdonald N.P., Lewis T., Guijt R.M., Paull B., Breadmore M.C. (2016). 3D printed microfluidic devices: Enablers and barriers. Lab Chip.

[62] Rogers C.I., Qaderi K., Woolley A.T., Nordin G.P. (2015). 3D printed microfluidic devices with integrated valves. Biomicrofluidics.

[63] Chan H.N., Shu Y., Xiong B., Chen Y., Chen Y., Tian Q., Michael S.A., Shen B., Wu H. (2016). Simple, Cost-Effective 3D Printed Microfluidic Components for Disposable, Point-of-Care Colorimetric Analysis. ACS Sens..

[64] Mathur A., Loskill P., Shao K., Huebsch N., Hong S., Marcus S.G., Marks N., Mandegar M., Conklin B.R., Lee L.P. (2015). Human iPSC-based Cardiac Microphysiological System For Drug Screening Applications. Sci. Rep..

[65] Toh Y.-C., Zhang C., Zhang J., Khong Y.M., Chang S., Samper V.D., Van Noort D., Hutmacher D.W., Yu H. (2007). A novel 3D mammalian cell perfusion-culture system in microfluidic channels. Lab Chip.

[66] Ong L.J.Y., Chong L.H., Jin L., Singh P.K., Lee P.S., Yu H., Ananthanarayanan A., Leo H.L., Toh Y.-C. (2017). A pump-free microfluidic 3D perfusion platform for the efficient differentiation of human hepatocyte-like cells. Biotechnol. Bioeng..

[67] Bian W., Bursac N. (2009). Engineered skeletal muscle tissue networks with controllable architecture. Biomaterials.

[68] Kwon Y.T., Kim Y.S., Kwon S., Mahmood M., Lim H.R., Park S.W., Kang S.O., Choi J.J., Herbert R., Jang Y.C. (2020). All-printed nanomembrane wireless bioelectronics using a biocompatible solderable graphene for multimodal human-machine interfaces. Nat. Commun..

[69] Kantaros A., Ganetsos T., Petrescu F.I.T., Ungureanu L.M., Munteanu I.S. (2024). Post-Production Finishing Processes Utilized in 3D Printing Technologies. Processes.

[70] Afshar M.E., Abraha H.Y., Bakooshli M.A., Davoudi S., Thavandiran N., Tung K., Ahn H., Ginsberg H.J., Zandstra P.W., Gilbert P.M. (2020). A 96-well culture platform enables longitudinal analyses of engineered human skeletal muscle microtissue strength. Sci. Rep..

[71] Kalman B., Picart C., Boudou T. (2016). Quick and easy microfabrication of T-shaped cantilevers to generate arrays of microtissues. Biomed. Microdevices.

[72] Kajtez J., Buchmann S., Vasudevan S., Birtele M., Rocchetti S., Pless C.J., Heiskanen A., Barker R.A., Martínez-Serrano A., Parmar M. (2020). 3D-Printed Soft Lithography for Complex Compartmentalized Microfluidic Neural Devices. Adv. Sci..

[73] Myalenko D., Fedotova O. (2023). Physical, Mechanical, and Structural Properties of the Polylactide and Polybutylene Adipate Terephthalate (PBAT)-Based Biodegradable Polymer during Compost Storage. Polymers.

[74] Shepherd R.F., Ilievski F., Choi W., Morin S.A., Stokes A.A., Mazzeo A.D., Chen X., Wang M., Whitesides G.M. (2011). Multigait soft robot. Proc. Natl. Acad. Sci. USA.

[75] Osaki T., Uzel S.G.M., Kamm R.D. (2020). On-chip 3D neuromuscular model for drug screening and precision medicine in neuromuscular disease. Nat. Protoc..

[76] Agrawal G., Aung A., Varghese S. (2017). Skeletal muscle-on-a-chip: An in vitro model to evaluate tissue formation and injury. Lab Chip.

[77] Iuliano A., Wal E., Ruijmbeek C., Groen S., Pijnappel W., de Greef J., Saggiomo V. (2020). Coupling 3D Printing and Novel Replica Molding for In House Fabrication of Skeletal Muscle Tissue Engineering Devices. Adv. Mater. Technol..

[78] Nikolic M., Sustersic T., Filipovic N. (2018). In vitro Models and On-Chip Systems: Biomaterial Interaction Studies With Tissues Generated Using Lung Epithelial and Liver Metabolic Cell Lines. Front. Bioeng. Biotechnol..

[79] Nikolakopoulou P., Rauti R., Voulgaris D., Shlomy I., Maoz B.M., Herland A. (2020). Recent progress in translational engineered in vitro models of the central nervous system. Brain.

[80] Stone N.L., England T.J., O’Sullivan S.E. (2019). A Novel Transwell Blood Brain Barrier Model Using Primary Human Cells. Front. Cell Neurosci..

[81] Dogan A.A., Dufva M. (2022). Customized 3D-printed stackable cell culture inserts tailored with bioactive membranes. Sci. Rep..

[82] Mc Carthy D.J., Malhotra M., O’Mahony A.M., Cryan J.F., O’Driscoll C.M. (2015). Nanoparticles and the Blood-Brain Barrier: Advancing from In-Vitro Models Towards Therapeutic Significance. Pharm. Res..

[83] Pohlit H., Bohlin J., Katiyar N., Hilborn J., Tenje M. (2023). Technology platform for facile handling of 3D hydrogel cell culture scaffolds. Sci. Rep..

[84] Booth R., Kim H. (2012). Characterization of a microfluidic in vitro model of the blood-brain barrier (μBBB). Lab Chip.

[85] Tan H.-Y., Trier S., Rahbek U.L., Dufva M., Kutter J.P., Andresen T.L. (2018). A multi-chamber microfluidic intestinal barrier model using Caco-2 cells for drug transport studies. PLoS ONE.

[86] Frost T.S., Jiang L., Lynch R.M., Zohar Y. (2019). Permeability of Epithelial/Endothelial Barriers in Transwells and Microfluidic Bilayer Devices. Micromachines.

[87] Huh D., Matthews B.D., Mammoto A., Montoya-Zavala M., Hsin H.Y., Ingber D.E. (2010). Reconstituting organ-level lung functions on a chip. Science.

[88] Rauti R., Ess A., Le Roi B., Kreinin Y., Epshtein M., Korin N., Maoz B.M. (2021). Transforming a well into a chip: A modular 3D-printed microfluidic chip. APL Bioeng..

[89] Qiu J., Gao Q., Zhao H., Fu J., He Y. (2017). Rapid Customization of 3D Integrated Microfluidic Chips via Modular Structure-Based Design. ACS Biomater. Sci. Eng..

[90] Leung C.M., de Haan P., Ronaldson-Bouchard K., Kim G.-A., Ko J., Rho H.S., Chen Z., Habibovic P., Jeon N.L., Takayama S. (2022). A guide to the organ-on-a-chip. Nat. Rev. Methods Primers.

[91] Fleck E., Keck C., Ryszka K., DeNatale E., Potkay J. (2023). Low-Viscosity Polydimethylsiloxane Resin for Facile 3D Printing of Elastomeric Microfluidics. Micromachines.

[92] Namgung H., Kaba A.M., Oh H., Jeon H., Yoon J., Lee H., Kim D. (2022). Quantitative Determination of 3D-Printing and Surface-Treatment Conditions for Direct-Printed Microfluidic Devices. BioChip J..

[93] Beckwith A.L., Borenstein J.T., Velasquez-Garcia L.F. (2018). Monolithic, 3D-Printed Microfluidic Platform for Recapitulation of Dynamic Tumor Microenvironments. J. Microelectromech. Syst..

[94] He Y., Xue G.H., Fu J.Z. (2014). Fabrication of low cost soft tissue prostheses with the desktop 3D printer. Sci. Rep..

[95] Tang C.K., Vaze A., Rusling J.F. (2017). Automated 3D-printed unibody immunoarray for chemiluminescence detection of cancer biomarker proteins. Lab Chip.

[96] Zhang L., Wang C., Zhang C., Xue Y., Ye Z., Xu L., Hu Y., Li J., Chu J., Wu D. (2024). High-Throughput Two-Photon 3D Printing Enabled by Holographic Multi-Foci High-Speed Scanning. Nano Lett..

[97] Hohmann J.K., Renner M., Waller E.H., von Freymann G. (2015). Three-Dimensional μ-Printing: An Enabling Technology. Adv. Opt. Mater..

